# Current challenges in carbon monoxide poisoning diagnosis from an analytical perspective

**DOI:** 10.3389/fmed.2023.1304294

**Published:** 2023-11-07

**Authors:** Stefania Oliverio

**Affiliations:** Forensic Toxicology Service, Department of Forensic Medicine, Laboratoire National de Santé, Dudelange, Luxembourg

**Keywords:** carbon monoxide poisoning, diagnosis, blood biomarker, measurement method, TBCO

## 1. Introduction

“Silent killer” is the most common nickname for carbon monoxide (CO). Not only is CO a colorless, odorless and tasteless molecule that is present in the atmosphere in gaseous form, but it is also a very toxic gas that is the leading cause of poisoning in many countries (1). Depending on the severity and duration of CO exposure, a variety of adverse health effects occur, such as headaches, nausea, cardiovascular dysfunctions and, in worst cases, death (2–4). CO is responsible for a cumulative mortality of 4.6 deaths per million and an incidence of 137 cases per million worldwide (5). Inhalation is the main mode of absorption of CO, which leads to a direct transfer into the bloodstream, where CO binds to hemoglobin (Hb). Due to the ~200–250 times higher affinity of Hb for CO than oxygen, CO competitively displaces oxygen, inhibiting oxygen transport and thus leading to hypoxia, which is the main toxic effect of CO (6). In addition, CO was shown to have direct cellular toxicity, which occurs through binding to other heme proteins such as myoglobin and cytochrome c oxidase, leading to skeletal muscle and myocardial toxicity as well as impaired oxidative metabolism (7, 8).

Diagnosis of CO poisoning occurs through evaluation of the symptoms and the case history by a clinician in the emergency department (ED) in combination with a confirmation through measurement of a biomarker. The main biomarker used for diagnosis confirmation is carboxyhaemoglobin (COHb) (9, 10). COHb is a direct biomarker of CO exposure, since it is formed directly after CO intake, and has a half-life of 4–6 h in blood (11). The measured COHb levels are used to give an indication of the level of CO exposure and can help determine the necessary treatment together with the symptoms reported by patients (12). However, there are several issues faced during CO poisoning diagnosis from both a clinical and analytical perspective.

## 2. Problems associated with CO poisoning diagnosis

### 2.1. CO intoxication severity

Depending on the levels of CO a person is exposed to and whether the exposure was acute or chronic, different symptoms may occur. Generally, symptomatology of CO is rather non-specific, since symptoms such as headaches, confusion, visual impairment and nausea are not associated to a specific disease but are rather general symptoms of illness (3, 13). This makes diagnosing CO poisoning in clinical settings very challenging, especially in cases where no suspicion of CO as the cause is present. However, in cases where CO is suspected as the cause of the illness, confirmation through measurement of blood biomarker COHb occurs (14). While in many cases the measured COHb concentration is in agreement with the symptoms and can lead to administration of the proper treatment for the patient, there is also a relatively high number of cases where measured COHb levels and reported symptoms are controversial.

Despite COHb being a direct biomarker of CO exposure, Often low COHb levels are measured in patients with CO exposure suspicion or with symptoms that are normally associated to higher COHb levels (13, 15, 16). This may lead to an erroneous diagnosis or treatment, which can have severe consequences. These discrepancies can be due to previously administered oxygen that reduced the COHb levels prior to hospital admission (3, 16, 17). While COHb levels in those cases have decreased significantly, symptoms and toxic effects of CO may still be increasing. Correct diagnosis and treatment in these cases is very difficult. If misdiagnosed, these cases can lead to delayed neurological sequelae (DNS) (18, 19). While mostly elevated COHb levels are necessary to produce DNS in acute exposure cases, DNS have also been reported in low-level chronic CO exposures (3, 20). These exposures are even more difficult to diagnose, since often CO exposure in these cases is not suspected and, even if measured, COHb levels are too low to be associated with CO poisoning. Nevertheless, chronic low-level CO exposure was also associated with a decrease in cognitive function and several neurological problems, some of them permanent (3, 21).

### 2.2. Current COHb measurement methods

Another important issue in CO poisoning diagnosis is related to the analytical measurement methods. CO can be measured in breath through a CO monitor, which measures the volume of CO in the end tidal breath in parts per million (ppm) and is correlated to blood COHb values (1, 22). This method is very useful for onsite measurement and is used by firefighters or paramedics for a rapid assessment. However, CO in breath does not represent the total amount of CO present in the body at the time of exposure and a high variability is often found due to the dependence on the breath-holding ability of the patient as well as other pulmonary characteristics (e.g., inspiration and expiration rates, capillary diffusion function, etc.) (23, 24).

In blood, COHb is measured either non-invasively through a pulse CO-oximeter, which determines the amount of CO bound to Hb through optical measurement (SpCO), or invasively by blood sampling and determination of COHb through blood gas analyser (25, 26). Despite the low-cost, non-invasiveness and time-efficiency in obtaining results, CO monitors and pulse-oximeters are known to have poor sensitivity, especially in the lower COHb concentration range, thus potentially leading to false negatives or falsely low COHb levels (27–29). One of the main reasons for the inaccurate measurements is the measurement principle of those monitors, which is spectrophotometry. Spectrophotometry is an optical measurement method, which measures the amount of COHb present in a sample by determining the amount of light absorbed at one or multiple specific wavelengths (30). While this method is very rapid and easy to use, it is prone to falsified results due to interferences that might be present in the blood sample (31, 32). An alternative method for COHb determination is the analysis through gas chromatography coupled to either a flame ionization detector (GC-FID) or mass spectrometer (GC-MS). The use of gas chromatographic methods gets rid of the issue of blood sample quality and potential interferences that affect spectrophotometric methods, giving more accurate and reliable results (33, 34). Nevertheless, GC methods are more time-consuming and costly and therefore, its current use in emergency medicine is almost non-existent; GC-MS is used mostly in post-mortem settings in forensic laboratories (14, 35–37).

### 2.3. Novel biomarker: total blood carbon monoxide (TBCO)

Another important aspect to consider for CO poisoning diagnosis is the fact that by using COHb as a biomarker, the principles behind the CO poisoning diagnosis are confined only to the effects caused by COHb alone. However, it is well-known that CO has direct toxic effects at cellular level, which are not due to the presence of COHb (38, 39). When using COHb as a biomarker, the effects caused by direct CO toxicity, such as impaired mitochondrial function, increased oxidative stress and inflammation in the brain, are not taken into account, thus potentially decreasing the diagnostic efficiency of COHb as a biomarker. Therefore, an alternative direct blood biomarker was investigated, total blood CO (TBCO) (40). The measurement of TBCO allows measurement of the total amount of CO present in the blood sample at the time of sampling, thus including both the CO bound to Hb but also the amount of free CO. The amount of free CO was estimated in previous studies (41, 42), but only in a recent study by the author, it was quantified for the first time. In a cohort of 13 patients, free CO was determined to vary between 20 and 80% of the TBCO, which is surprisingly more than previous studies had suggested (40). These differences are quite substantial and could potentially explain the discrepancies between COHb levels and symptoms reported by patients in some cases. Determining the total amount of CO seems to be more in line with the pathophysiology of CO poisoning. Furthermore, TBCO could improve CO poisoning diagnosis by improving accuracy and sensitivity, thus reducing the likelihood of misdiagnosis, even in the more challenging cases. Nevertheless, it should be noted that the study cohort was very limited; therefore, further studies need to be carried out to confirm these results. As opposed to COHb, TBCO is currently analyzed by GC-MS only (40, 43).

## 3. Discussion

Diagnosing CO poisonings is a challenging task for clinicians due to the non-specificity of the associated symptoms, which make it difficult to associate a case to CO poisoning by the symptoms alone (44). Usually, the patient's history, which might include a known source of exposure to CO, such as a fire or gas leak, can help in endorsing the resulting illness. Confirmation is then obtained by measurement of a blood biomarker (45). Several biomarkers for CO exposure were investigated, which were mostly indirect biomarkers, such as lactate or serum bilirubin levels (46, 47). Indirect biomarkers are usually easy to measure, but known to be altered by several diseases or genetic factors, thus not having high specificity (46–48). Therefore, the main blood biomarker for CO poisoning is COHb (45). In clinical settings, COHb is measured by pulse-oximetry or blood gas analysers, which have the advantage of being easy to use, cheap and quick; as opposed to the measurement via gas chromatography, which is more time-consuming, but gives results that are more accurate (32). The main disadvantage of using COHb as a biomarker is that it does not fully account for the toxicodynamic effects of CO, it limits the diagnostic principle to the CO bound to Hb. This is not in accordance with the known pathophysiology of CO poisoning (8, 12).

Therefore, an alternative direct CO biomarker was investigated, which is TBCO. TBCO measures the amount of both CO bound to Hb and of the free CO present in blood (40). This biomarker seems to be able to give a more complete picture of the case at hand, since it can account not only for the hypoxic effects caused by the formation of COHb, but also the CO toxicity occurring at cellular level. A previous study showed differences of TBCO compared to COHb that can vary from 20 to 80%, which can significantly change the therapeutic strategy for patients and potentially reduce the cases where CO poisoning is misdiagnosed or the diagnosis is delayed. DNS and other long-term effects can be the result of these misdiagnoses (49). But DNS can also result from low-level chronic exposures, which are even more difficult to associate to CO exposure due to the delay in appearance of the symptoms, but also the low sensitivity of available measurement methods for COHb (32). The use of TBCO as a biomarker could therefore improve diagnosis of these low-level chronic exposures, given the higher measurement accuracy.

One disadvantage of TBCO measurement is that it is analyzed via GC-MS only, which is a method that is currently not readily available in many laboratories in emergency medicine. However, it is present in most university hospital laboratories as well as forensic laboratories, thus requiring only a collaboration with a neighboring laboratory. Measurement of TBCO with other more quick and cheap measurement techniques, such as spectrophotometry, can be investigated, with the remaining constraint of sample quality. The limited number of study subjects as well as the higher time and costs involved in the measurement of TBCO are additional limitations for this biomarker. Further studies are necessary to confirm the accuracy of TBCO as a more complete biomarker for CO poisoning diagnosis, ideally with studies aimed at sampling blood as close as possible to the time of exposure, either directly at the scene or in the ambulance, and comparing the results for COHb and TBCO in these cases. Getting a better picture of the differences between COHb and TBCO might better elucidate the mechanisms of CO poisonings and enable a more precise treatment for patients. The complexity of CO pathophysiology requires more research, but important steps have been taken and should be investigated further to be able to reduce the many challenges in CO poisoning diagnosis.

## Author contributions

SO: Conceptualization, Methodology, Project administration, Writing—original draft, Writing—review & editing.
